# Habitat Diversity Sustains Ecosystem Functioning in Plateau Arid‐Region Wetlands

**DOI:** 10.1002/ece3.72747

**Published:** 2025-12-17

**Authors:** Chenyu Wang, Haicheng Wei, Ronglei Duan, Shi Jin, Jinxin Wen, Hongyu Li, Aiying Cheng, Chunliang Gao, Hongpan Xue, Yilin Hou

**Affiliations:** ^1^ Key Laboratory of Green and High‐End Utilization of Salt Lake Resources Qinghai Institute of Salt Lakes, Chinese Academy of Sciences Xining China; ^2^ Qinghai Provincial Key Laboratory of Geology and Environment of Salt Lakes, Qinghai Institute of Salt Lakes Chinese Academy of Sciences Xining China; ^3^ University of Chinese Academy of Sciences Beijing China; ^4^ Center of Deep‐Sea Research, Institute of Oceanology Chinese Academy of Sciences Qingdao China

**Keywords:** arid‐region, biogeochemical cycles, habitat diversity, metagenomics, Qinghai‐Tibet plateau, wetlands

## Abstract

Plateau arid‐region wetlands constitute critical ecosystems for regional ecological security, yet exhibit heightened vulnerability under multiple stressors. Current understanding of the mechanisms sustaining the functions of these systems, particularly the pivotal role of habitat diversity, remains limited. Targeting the Jinzihai Wetland (Qaidam Basin, Qinghai‐Tibet Plateau), we integrated metagenomic and geochemical profiling to characterize three representative habitats: sandy meadows, peat bogs, and lake sediments. Our analyses revealed that pronounced cross‐habitat physicochemical gradients drive community structure differentiation predominantly through species replacement, establishing habitat diversity as a fundamental driver of wetland biodiversity. Concurrently, community differentiation drives spatial divergence in functional gene composition, manifesting distinct functional dominance: sandy meadows govern assimilation and saline‐alkaline stress response; peat bogs orchestrate nutrient enrichment and transformation; lake sediments mediate element release and burial. These functionally complementary habitats collectively catalyze biogeochemical cycling. We demonstrate that within plateau arid‐region wetlands, habitat diversity stabilizes ecosystem functioning by sustaining both biodiversity and functional diversity of biogeochemical processes. Consequently, prioritizing habitat diversity conservation is imperative for safeguarding the long‐term stability of these vulnerable ecosystems within management frameworks.

## Graphical abstract

Habitat diversity underpins both biodiversity and functional biogeochemical processes in plateau arid‐region wetlands, thereby stabilizing ecosystem functioning. These functionally complementary habitats collectively drive critical ecosystem processes. Consequently, conserving habitat diversity is essential for maintaining the long‐term stability of these vulnerable ecosystems.

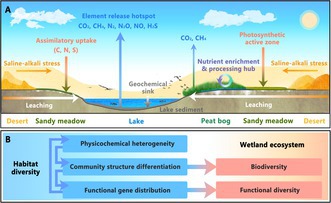

## 1 Introduction

Wetlands constitute globally vital ecosystems, delivering irreplaceable services in climate regulation, environmental purification, and biodiversity maintenance (Qu et al. 2024; Zou et al. 2022; Simpkins 2021; Kang et al. 2020). Yet, under mounting pressures from climate change and anthropogenic activities, these ecosystems are degrading at an unprecedented pace, ranking among the fastest‐disappearing ecosystems globally (Kundu et al. 2024; Qu et al. 2024; Moi et al. 2022; Xu et al. 2019). The Qinghai‐Tibet Plateau (QTP), renowned as the “Asian Water Tower”, hosts wetlands covering over 100,000 km^2^, accounting for approximately one‐third of China's total wetland area (Wei et al. 2015; Niu et al. 2009). Crucially, arid/semi‐arid climate regions cover more than half of the QTP's area yet harbor extensive wetlands (Wu et al. 2003). These wetlands are strategically indispensable: they underpin regional endemic biodiversity, maintain pivotal biogeochemical cycles, and safeguard ecological security and human livelihoods across surrounding landscapes. Nevertheless, synergistic stressors including intense evaporation, precipitation scarcity, chemical contamination, and hydrological fluctuations significantly amplify degradation risks, heightening their ecological vulnerability (Cheng et al. 2024; Liu et al. 2024; Fu et al. 2024; Jin et al. 2013). Consequently, elucidating the intrinsic mechanisms stabilizing plateau arid‐region wetlands amidst climate change is paramount for developing science‐based conservation strategies to secure the QTP's ecological integrity.

Habitat diversity is increasingly acknowledged as a critical factor influencing the stability of wetland ecosystems. It supports ecosystem functioning and long‐term resilience by mediating environmental heterogeneity, species richness, structural complementarity, and key ecological processes (Liu et al. 2024; Alsterberg et al. 2017; Engelhardt and Ritchie 2001). However, contemporary research on wetlands in arid regions of the QTP remains predominantly centered on lake systems (Li et al. 2025; Cheng et al. 2024; Wang et al. 2018). The prevailing research paradigm often treats complex wetland ecosystems as homogeneous entities, largely restricted to investigating single habitat types or taxonomic groups through methodologies dominated by chemical analysis and remote sensing (Fu et al. 2024; Cheng et al. 2024; Shi et al. 2024; Xue et al. 2018). While such studies characterize macro‐scale environmental patterns, understanding of the mechanisms sustaining ecosystem functioning, particularly the central role of habitat diversity, remains critically deficient. This knowledge gap stems from two interrelated constraints: (1) Insufficient quantitative characterization and differentiation of key physicochemical gradients (e.g., moisture, metals, organic matter) between distinct habitats (e.g., meadows, bogs, lakes) within wetlands, constraining comprehension of intra‐wetland habitat heterogeneity; (2) Scarce research examines how differences in physicochemical properties among habitats drive biological community assembly and shape biogeochemical cycling functions in plateau arid‐region wetlands through the lens of microbial communities and functional genes. These deficiencies collectively hinder the development of effective conservation frameworks for plateau arid‐region wetlands.

To address these gaps, this study focuses on Jinzihai, a representative arid‐region wetland in the Qaidam Basin on the QTP. We investigate three core habitats: sandy meadows (SM, characterized by saline‐alkaline and inorganic conditions), peat bogs (PB, water‐saturated and organic‐rich), and lake sediments (LS, anoxic and reducing). By integrating metagenomic profiling of microbial communities with geochemical analysis, we simultaneously quantify spatial heterogeneity in environmental gradients and resolve the community structure and functional gene networks (the associations among genes) governing carbon (C), nitrogen (N), and sulfur (S) cycling. This methodology bridges geochemical and metagenomic datasets to systematically quantify the environmental matrix and elucidate the supported biological communities, thereby establishing mechanistic linkages across scales. Accordingly, we addressed two central questions to elucidate the mechanisms by which habitat diversity sustains ecosystem functioning in plateau arid‐region wetlands: (1) How do physicochemical variations among habitats shape microbial community composition and functional gene distribution? (2) How does habitat diversity influence the functional diversity of biogeochemical cycling processes? By deciphering these genomic and biogeochemical linkages, we establish the conservation imperative of habitat diversity for these vulnerable landscapes. Our findings provide the foundational framework for adaptive management of wetlands, with profound implications for the ecological integrity of the “Asian Water Tower”.

## 2 Materials and Methods

### 2.1 Study Area and Sampling

**FIGURE 1:**
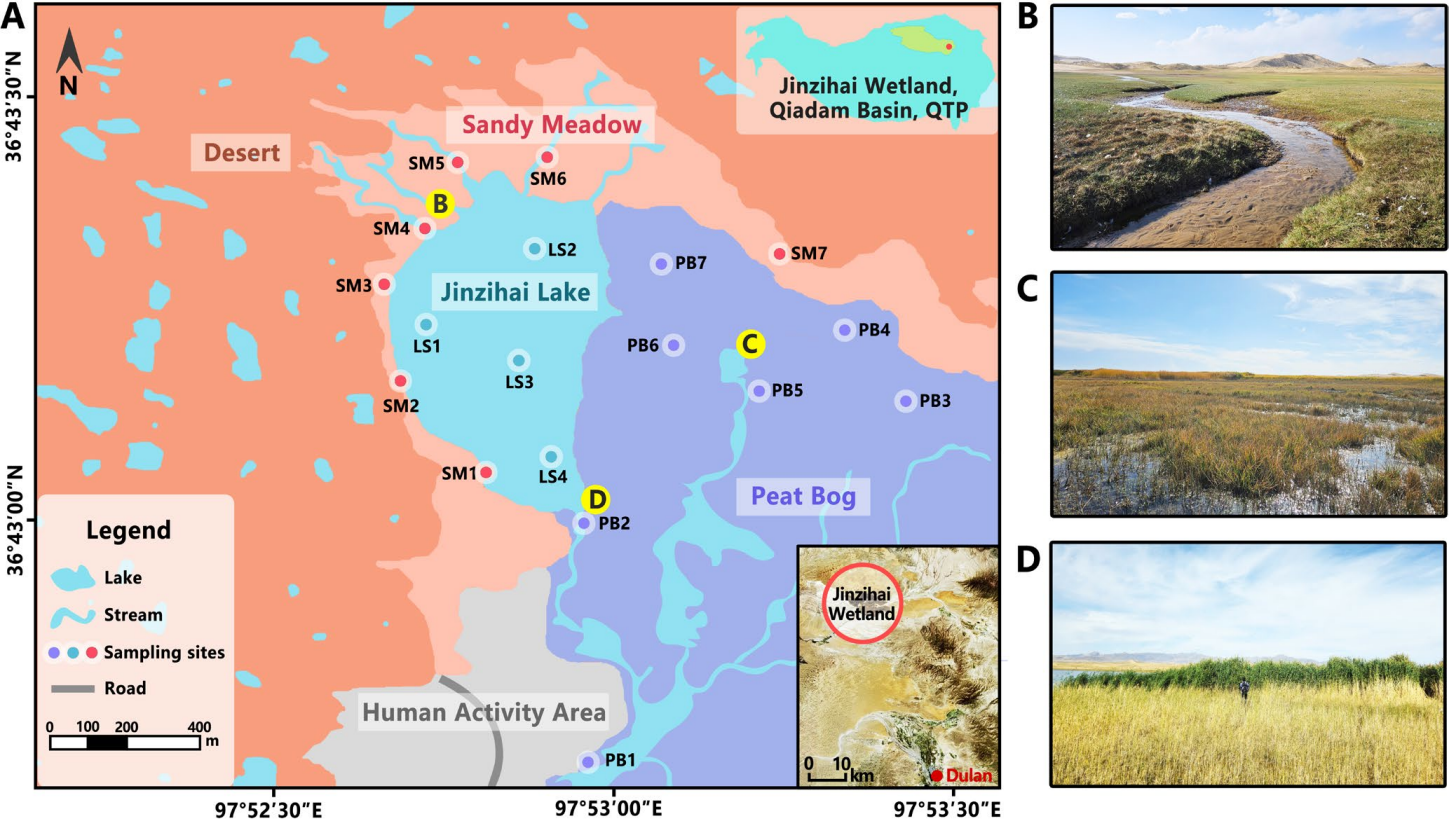
Study area overview. (A) Distribution of sampling sites, including topsoil samples from sandy meadows (SM) and peat bogs (PB), and lake sediments (LS) samples. A satellite image (from Jilin‐1, 2024) illustrates the spatial relationship between the Jinzihai Wetland and the nearest town (Dulan). Detailed GPS coordinates for each site are provided in Table A1. Field photographs show: (B) sandy meadow and (C, D) peat bog.

Jinzihai Wetland (36°40′–36°44′ N, 97°49′–97°59′ E, 2978–3012 m), covering an area of ~50 km^2^, is located in the eastern Qaidam Basin of the QTP (Figure 1A). Climatic data from multi‐site averages across the Qaidam Basin reveal a mean annual precipitation of only ∼100 mm and an annual evaporation of 2000 mm (Zeng et al. 2024). As the nearest meteorological station, Dulan Station (36°18′ N, 98°06′ E) records a mean annual precipitation of 218 mm and a mean annual temperature of 3.4°C. These meteorological conditions demonstrate that Jinzihai is characteristic of a typical plateau arid‐region wetland. The wetland comprises numerous small lakes, with Jinzihai Lake (~0.23 km^2^) serving as the primary waterbody. The lakebed is composed of permanently submerged sediments that support abundant growth of Potamogetonaceae. The lakeshore exhibits distinct habitat heterogeneity: the northwestern shore is supplied by several surface streams originating from the adjacent desert, forming sandy meadows in the desert‐lake ecotone. This habitat is dominated by Arenosols (IUSS Working Group WRB 2015), with vegetation consisting mainly of Asteraceae, Caprifoliaceae, and Cyperaceae (Figure 1B). In contrast, the southeastern shore consists of extensive peat bogs, which are primarily sustained by groundwater discharge. These bogs are characterized by Histosols (IUSS Working Group WRB 2015), with a vegetation cover dominated by Cyperaceae, Poaceae, Characeae, and Sphagnaceae (Figure 1C,D). Runoff from these bogs combines with the lake outflow to feed the Sulengguole River, which ultimately discharges into Beihuobuxun Salt Lake. The wetland sustains rich faunal diversity, including rare species such as *Anser cygnoides* , *Felis silvestris bieti*, and *Grus nigricollis* , reflecting its significant ecological value.

Field sampling was conducted in September 2024. Topsoil samples (0–10 cm depth) were collected from sandy meadows and peat bogs using stainless‐steel shovels, with seven sites per habitat. Surface sediments were collected from four sites in Jinzihai Lake using a grab sampler. During sampling, nitrile gloves and masks were worn to minimize contamination. All tools were either replaced or cleaned with anhydrous ethanol and sterile water between sample collections. At each site, three replicate samples (collected at ~1 m intervals) were combined into one sterile bag to form a composite sample. These composites were thoroughly homogenized and subsampled: portions for metagenomic analysis were transferred into cryotubes, flash‐frozen on dry ice, and transported to the biological laboratory; the remaining subsamples were refrigerated and transported to the chemical laboratory for physicochemical analysis.

### 2.2 Physicochemical Analysis of Samples

We conducted multiple physicochemical analyses on soil/sediment samples. Soil water content (SWC) was determined by oven‐drying 10 g of fresh sample at 105°C to constant weight. pH was measured using a pH meter (PHS‐3E, INESA, China): soil/sediment were mixed with water at a 2:5 mass ratio. Total carbon (TC), total nitrogen (TN), and total sulfur (TS) were measured using an elemental analyzer (Vario EL Cube, Elementar, Germany). A Continuous Flow Analyzer (SAN++, Skalar, Holland) determined total dissolved nitrogen (TDN), ammonium nitrogen (NH_4_^+^‐N), nitrate nitrogen (NO_3_^−^‐N), and total phosphorus (TP). Sulfate (SO_4_^2−^) was measured by ICP‐AES (Avio550max, PerkinElmer, USA). Metal elements (K, Ca, Ba, Mn, Fe) were analyzed using an X‐ray fluorescence spectrometer (Vanta, Innov‐X System, USA). Total organic carbon (TOC) was quantified using the elemental analyzer on acid‐leached samples (1 mol/L HCl, 48 h, washed). Carbon pools were assessed via a two‐step acid hydrolysis (Rovira and Vallejo 2002): 0.5 g sieved sample was hydrolyzed with 20 mL 2.5 mol/L H_2_SO_4_ at 105°C for 30 min. After centrifugation and washing, the supernatant formed labile carbon pool I (LCP1). The residual solid was dried, treated with 2 mL 13 mol/L H_2_SO_4_ at 25°C for 12 h, diluted to 2 mol/L H_2_SO_4_, and hydrolyzed at 105°C for 3 h. The subsequent supernatant constituted labile carbon pool II (LCP2). The final dried residue comprised the recalcitrant carbon pool (RCP). LCP1&2 were quantified using a TOC analyzer (TOC‐LCPH, Shimadzu, Japan). RCP was measured using the elemental analyzer. Original data are provided in Table A2.

### 2.3 Metagenome Sequencing and Analysis

Total DNA was extracted from soil/sediment samples using the E.Z.N.A. Soil DNA Kit (Omega Bio‐tek, USA). Extracted DNA was fragmented using a Focused‐ultrasonicator (M220, Covaris, USA). Sequencing libraries were prepared using the TruSeq DNA Library Preparation Kit (Illumina, USA), quantified with a Qubit Fluorometer (Thermo Fisher Scientific, USA), and sequenced paired‐end (PE150) on the Illumina NovaSeq 6000 platform by Biozeron Biological Technology Co. Ltd. (Shanghai, China), yielding ~10 Gb per sample.

Raw sequence data underwent quality control and adapter trimming using Trimmomatic (Bolger et al. 2014). Clean reads were generated by aligning to the human genome with BWA‐MEM (http://bio‐bwa.sourceforge.net/bwa.shtml) and removing human‐derived sequences. De novo assembly of clean reads was performed using MEGAHIT (v1.1.1) to generate contigs (Li et al. 2015). Open reading frames (ORFs) within contigs were predicted using Prodigal (v2.6.3) (Hyatt et al. 2010). Predicted genes were clustered (95% identity, 90% coverage) with CD‐HIT (v4.8.1) to create a non‐redundant gene catalog (Fu et al. 2012). Gene abundance was quantified using Salmon (Patro et al. 2017). Taxonomic annotation of non‐redundant genes was achieved via BLASTP searches against the NCBI non‐redundant protein database. Functional annotation involved querying non‐redundant protein sequences against CCycDB (https://ccycdb.github.io/), NCycDB (https://github.com/qichao1984/NCyc), SCycDB (https://github.com/qichao1984/SCycDB), and EggNOG (http://eggnog6.embl.de/) databases using DIAMOND (Zhou 2023; Yu et al. 2021; Tu et al. 2019; Huerta‐Cepas et al. 2019; Buchfink et al. 2015). Carbohydrate‐active enzymes (CAZymes) were annotated by matching protein sequences against the hidden Markov model (HMM) libraries of CAZyme families retrieved from the CAZy Database (http://www.cazy.org/), using HMMER (v3.2.1) (Lombard et al. 2014; Eddy 2011). Kyoto Encyclopedia of Genes and Genomes (KEGG) ortholog annotation was performed using KofamScan (Kanehisa et al. 2025; Potter et al. 2018). Detailed database annotation results are provided in Data S1.

### 2.4 Statistical Analysis

**FIGURE 2:**
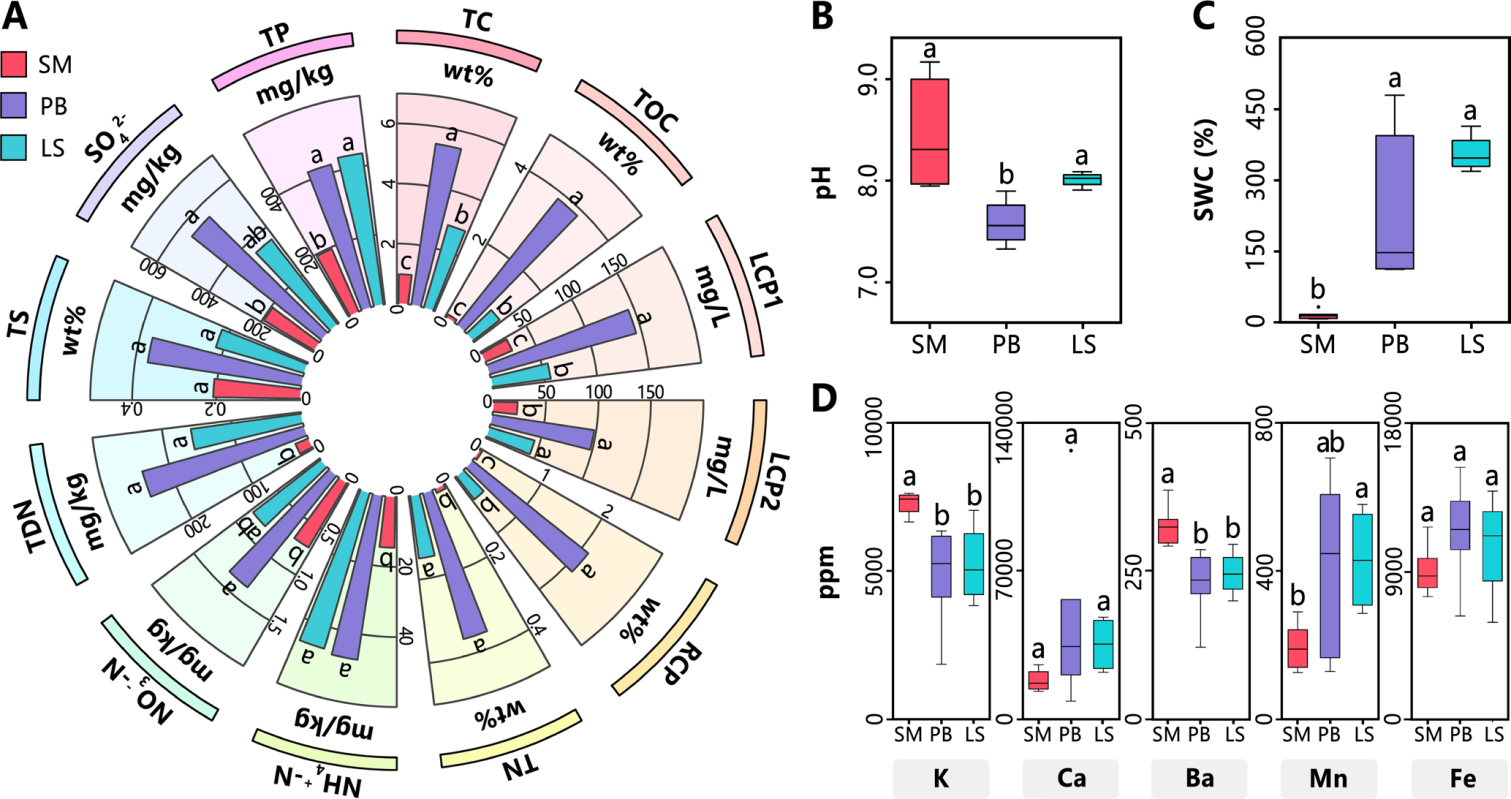
Element distribution in Jinzihai Wetland. For SM, PB, and LS habitats: (A) Concentrations and differences of C, N, S, P compounds (column height = median). (B, C) Differences in pH and SWC. (D) Concentrations and differences of metal elements. Central line and whiskers in each box represent the median and the min‐max values; boxes indicate interquartile range (25th–75th percentile). Different letters denote significant inter‐group differences (determined by two‐sided Wilcoxon test, FDR‐adjusted *p* < 0.05).

**FIGURE 3:**
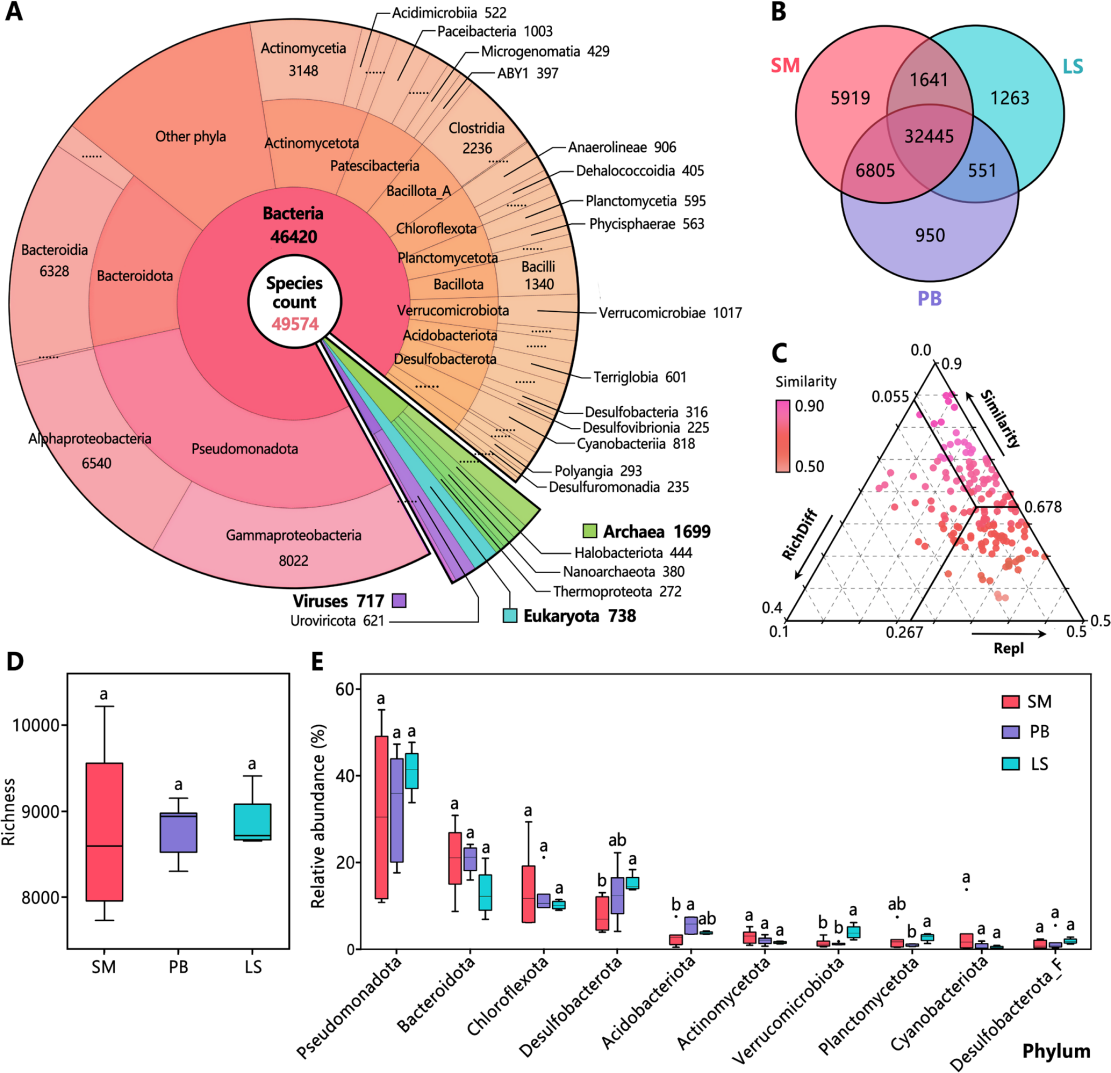
Characteristics of biodiversity and community structure in Jinzihai Wetland. (A) Species taxonomic annotation based on metagenomic data. (B) Unique and shared species among SM, PB, and LS habitats. (C) β‐diversity decomposition among samples. Jaccard dissimilarity (1‐Similarity) decomposed into replacement (Repl) and richness difference (RichDiff) components. (D) Differences in species richness among habitats. (E) Relative gene abundance of top 10 phyla and inter‐habitat differences, only the top 10 phyla are displayed. Central line and whiskers in each box represent the median and the min‐max values, boxes indicate interquartile range (25th‐75th percentile). Single points are outliers. Different letters denote significant inter‐group differences (determined by two‐sided Wilcoxon test, FDR‐adjusted *p* < 0.05).

**FIGURE 4:**
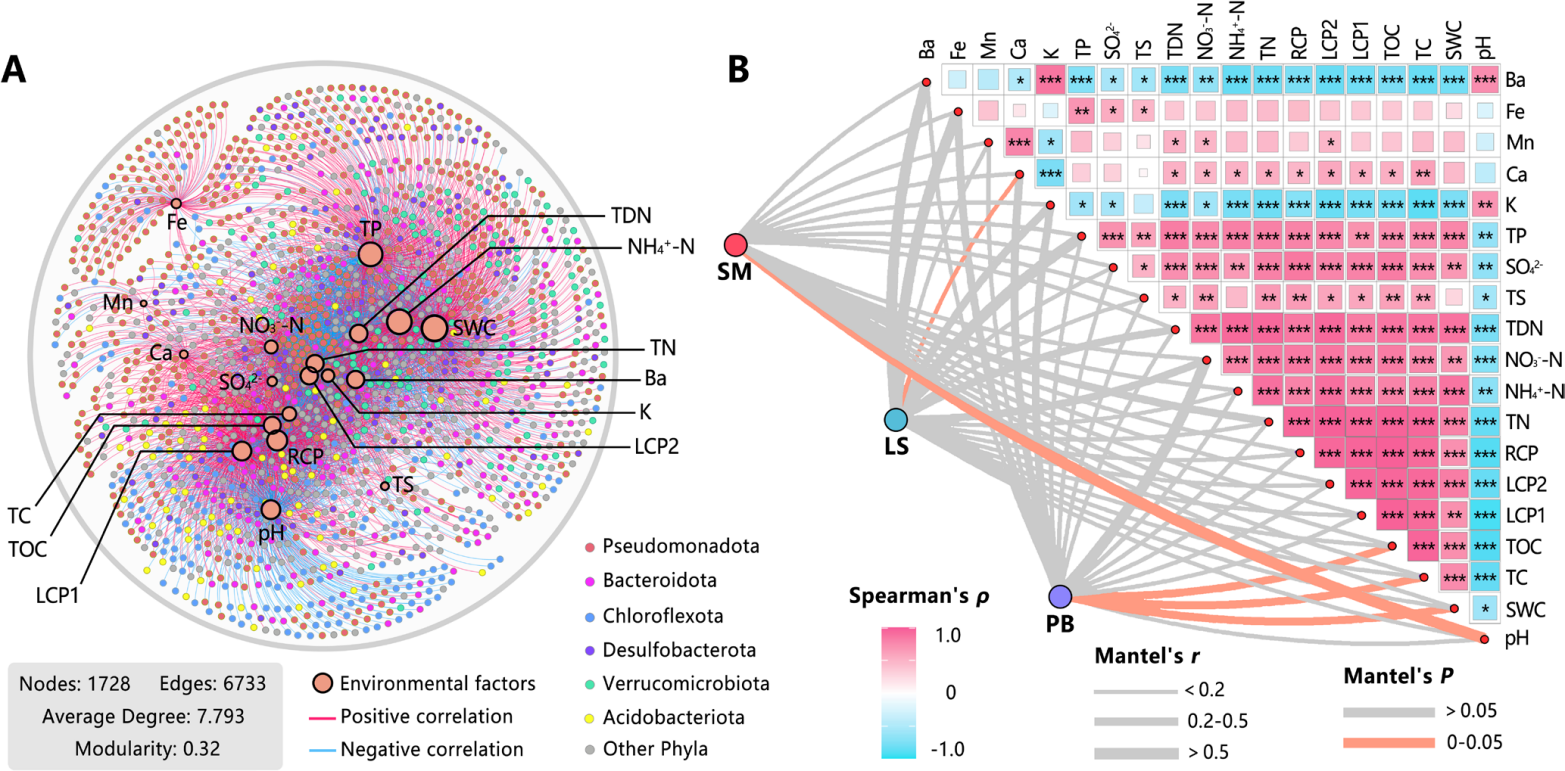
Associations between environmental factors and communities in Jinzihai Wetland. (A) Co‐occurrence network between environmental factors and species. Nodes representing species are colored by phylum. Edges indicate significant correlations (Spearman's |*ρ*| > 0.6, FDR‐adjusted *p* < 0.05), with color denoting the direction of the correlation. (B) Partial Mantel test associations between community and environmental factors (edge width = Mantel's *r*, edge color = significance). Heatmap colors represent Spearman's *ρ* correlation coefficients; significance levels are denoted as *p < 0.05, **p < 0.01, ***p < 0.001.

Statistical analyses integrated physicochemical parameters and taxonomic/functional gene annotation abundances. Physicochemical differences between habitats were visualized using bar/box plots and assessed via two‐sided Wilcoxon tests (FDR‐adjusted *p* < 0.05; Figure 2A–D). Subsequently, taxonomic composition for the wetland was visualized using Krona (Figure 3A) (Ondov et al. 2011), and Venn diagrams depicting unique/shared species among habitats (Figure 3B). Alpha diversity was evaluated based on species richness. Inter‐group differences in species richness and relative abundance of genes annotated to the top 10 phyla were visualized using box plots and assessed by two‐sided Wilcoxon tests (FDR‐adjusted *p* < 0.05; Figure 3D,E). Furthermore, Jaccard dissimilarity (1‐similarity) between all samples was computed using the *beta.div.comp* function from the *adespatial* R package (Dray et al. 2012). This dissimilarity was decomposed into Replacement (Repl) and Richness Difference (RichDiff) components (Legendre 2014). Mean Similarity, Repl, and RichDiff values were computed to examine β‐diversity sources (Figure 3C). To identify key taxa driving differentiation across the three habitats, we performed LEfSe analysis on the four domains (Archaea, Bacteria, Eukaryota, and Viruses) using the *microeco* package (Liu et al. 2025). During the analysis of each domain, an “others” category (sum of all other domains) was incorporated to normalize all taxonomic unit abundances to the total community, thereby eliminating bias from domain‐specific analysis. Results are shown in Figures A4, A5, A6, A7 (highlighting taxa based on high abundance and high LDA scores, details in figure captions). To evaluate the relationships between environmental factors and community structure (based on Bray‐Curtis distance), partial Mantel tests were performed using the *mantel.partial* function from the *vegan* package (Oksanen et al. 2025; Borcard and Legendre 2012). Meanwhile, Spearman correlations among the environmental factors were calculated using the *correlate* function from the *ggcor* package (Huang et al. 2020). Results from the partial Mantel tests and Spearman correlations were visualized using the *linkET* package (Kang et al. 2024; Huang 2021) (Figure 4B). Additionally, a co‐occurrence network linking environmental factors and species was constructed using Gephi (v0.10) (Bastian et al. 2009) (Figure 4A). Species nodes were included if they occurred in > 50% of samples with a cumulative relative gene abundance > 0.001%. Edges represent significant correlations (Spearman |*ρ*| > 0.6 and FDR‐adjusted *p* < 0.05).

**FIGURE 5:**
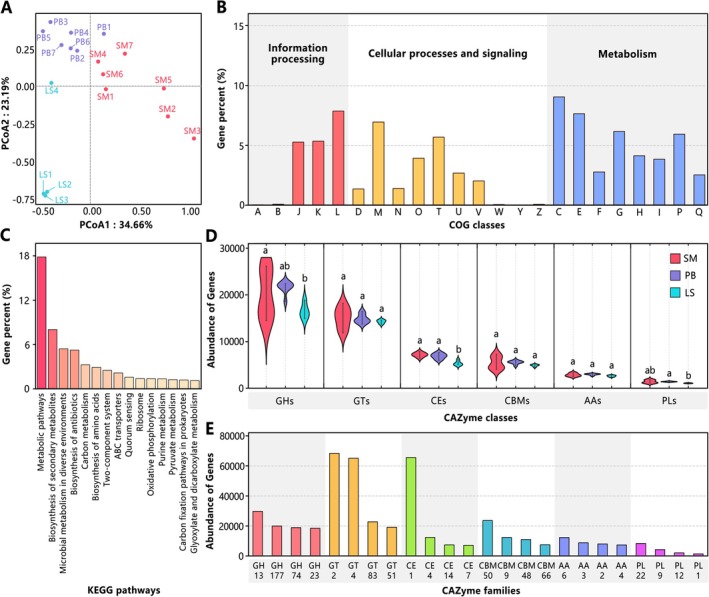
Functional annotation of Jinzihai Wetland samples metagenomic contigs. For SM, PB, and LS habitats: (A) PCoA based on gene abundance. (B) COG class distribution. A‐Z are as described by the EggNOG database (http://eggnog6.embl.de/download/eggnog_4.5/COG_functional_categories.txt). (C) Relative abundance of the top 15 KEGG pathways. (D) CAZyme class annotations and significant inter‐group differences. Classes: Glycoside hydrolases (GHs), glycosyl transferases (GTs), polysaccharide lyases (PLs), carbohydrate esterases (CEs), auxiliary activities (AAs), and carbohydrate‐binding modules (CBMs). Violin plots show min‐max (whiskers) and kernel density (width). Different letters denote significant inter‐group differences (determined by two‐sided Wilcoxon test, FDR‐adjusted *p* < 0.05). (E) Summary of annotated CAZyme families across all samples. The top 4 families within each class are presented.

**FIGURE 6:**
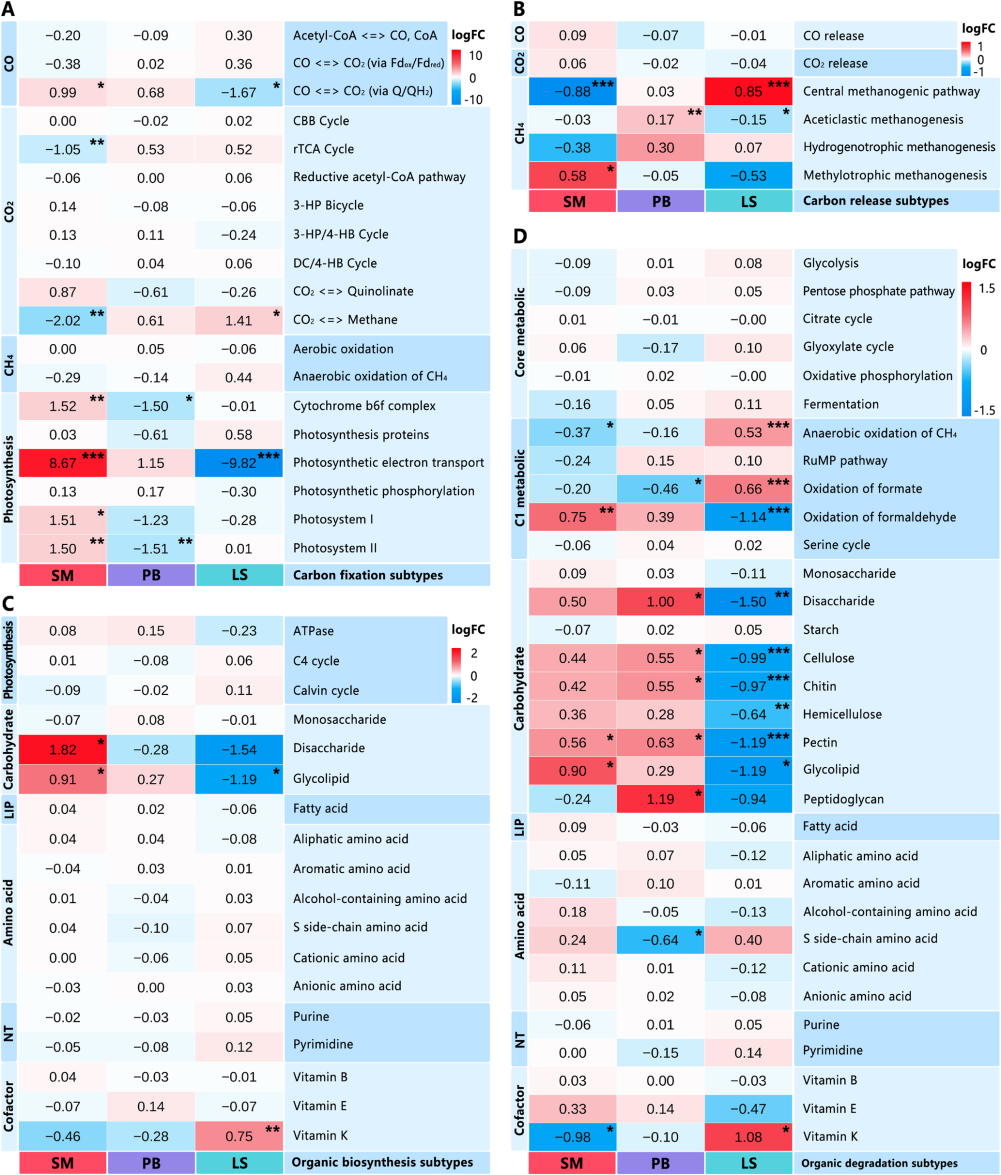
Functional annotation of carbon cycle in metagenomic contigs from Jinzihai Wetland samples. (A) Carbon fixation subtypes, (B) Carbon release subtypes, (C) Organic biosynthesis subtypes, and (D) Organic degradation subtypes. The heatmap illustrates enrichment differences among the SM, PB, and LS habitats. *P*‐values were derived from two‐sided likelihood ratio tests (LRTs) and adjusted for multiple comparisons using the Benjamini‐Hochberg false discovery rate (FDR) procedure (*p < 0.05, **p < 0.01, ***p < 0.001). LogFC denotes log2‐fold change.

**FIGURE 7:**
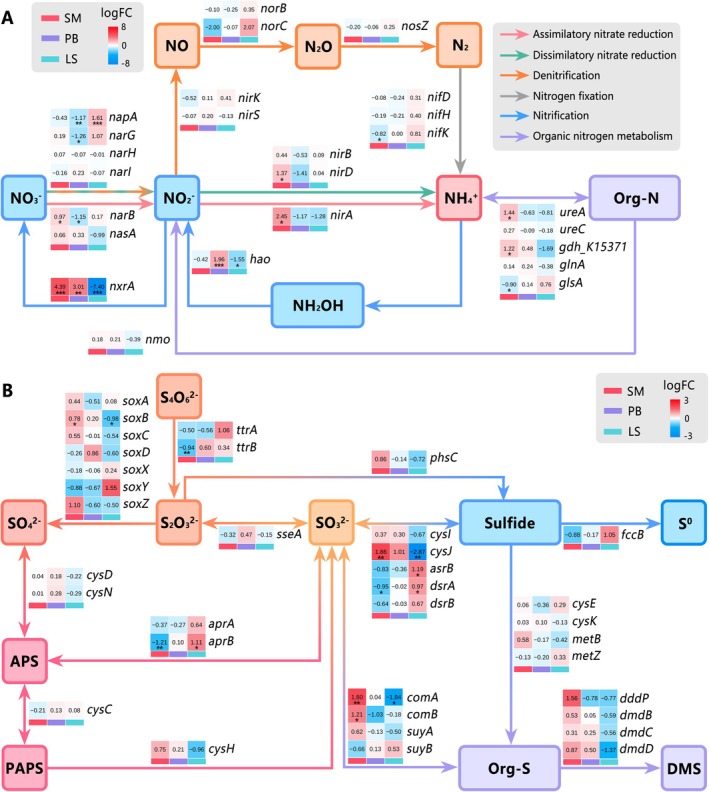
Differential enrichment profiles of nitrogen/sulfur cycle functional genes across habitats in Jinzihai Wetland. (A) Nitrogen cycling and (B) Sulfur cycling. *p*‐values were derived from two‐sided likelihood ratio tests (LRTs) and adjusted for multiple comparisons using the Benjamini‐Hochberg false discovery rate (FDR) procedure (*p < 0.05, **p < 0.01, ***p < 0.001). LogFC denotes log2‐fold change.

Principal Coordinates Analysis (PCoA) based on Bray‐Curtis distances visualized overall gene compositional differences among samples (Figure 5A). Functional gene profiles were characterized by displaying TPM‐normalized abundances of genes annotated to COG classes, KEGG pathways, and CAZyme classes/families using bar/violin plots (Figure 5B–E) (Huerta‐Cepas et al. 2019; Kanehisa et al. 2025; Lombard et al. 2014), with inter‐group differences assessed by two‐sided Wilcoxon tests (FDR‐adjusted *p* < 0.05). For the carbon cycle, genes were categorized into carbon fixation/release and organic biosynthesis/degradation subtypes based on CCycDB (Zhou 2023), with inter‐habitat differential enrichment visualized via heatmaps (Figure 6A–D). For nitrogen and sulfur cycles, process flowcharts were constructed based on NCycDB and SCycDB annotations (Yu et al. 2021; Tu et al. 2019), supplemented with heatmaps highlighting differential enrichment of key functional genes among habitats (Figure 7A,B). Differential enrichment analysis was performed using raw read counts as input and assessed using the following analytical pipeline implemented in the *edgeR* package (Robinson et al. 2010): lowly expressed genes were filtered out using the *filterByExpr* function (min.total.count = 10, min.prop = 0.2) (Chen et al. 2016); normalization was then performed using the *calcNormFactors* function with the TMM method (Robinson and Oshlack 2010); dispersion was estimated using the *estimateDisp* function (Datta and Nettleton 2014); finally, a generalized linear model (GLM) assuming a negative binomial distribution was fitted to the data using the *glmFit* function to identify differentially enriched genes (McCarthy et al. 2012), detailed results are available in Data S2.

## 3 Results

### 3.1 Physicochemical Gradients Across Multi‐Habitats in Jinzihai Wetland

Significant differences were observed in the physicochemical properties of surface substrates among the three primary habitats of Jinzihai Wetland: sandy meadow (SM), peat bog (PB), and lake sediment (LS) (Figure 2A–D). SM exhibited the lowest SWC (*p* < 0.05), an alkaline pH (7.95–9.17), and high inorganic carbon content (median 92%) (Table A2). Contents of TC, TOC, LCP1, LCP2, RCP, TN, TDN, NH_4_^+^‐N, and TP in SM were all significantly lower than those in PB and LS (*p* < 0.05). For metallic elements, SM showed significant enrichment in Ba and K (*p* < 0.05) but lower Fe and Mn contents. PB displayed high SWC (median 146.5%) and near‐neutral pH (7.33–7.90). Its TC, TOC, LCP1, RCP, TN, TDN, NH_4_^+^‐N, NO_3_^−^‐N, SO_4_^2−^, and TP contents were all significantly higher than those in SM (*p* < 0.05). Notably, site PB2, on the shore of the lake inlet, exhibited extremely high SWC (479.0%), along with high TOC (16.08%), TN (1.81%), TS (0.86%), and TP (0.092%). LS was weakly alkaline (pH 7.91–8.03). Concentrations of TC, TOC, RCP, SO_4_^2−^, and TP were intermediate between SM and PB. Metallic elements in LS were enriched in Mn and Fe. Elemental correlation analysis revealed positive associations between SWC and C, N, P, S components but negatively with K, Ba, and pH (Figure 4B). Furthermore, weaker positive correlations were also observed between SWC/C/N/P/S and Ca/Mn/Fe.

### 3.2 Community Differentiation Among Multi‐Habitats in Jinzihai Wetland

Metagenomic annotation identified 49,574 species spanning Bacteria, Archaea, Eukaryotes, and Viruses (Figure 3A). Analysis of shared and unique species showed numerous non‐shared species (17,129) across the three habitats (Figure 3B). The terrestrial habitats SM and PB shared the most species (39,250), while SM contained the highest number of unique species (5919). Alpha diversity analysis indicated no significant differences in species richness among habitats (Figure 3D). β‐diversity decomposition revealed a high replacement index (Repl = 0.267) and a low richness difference index (RichDiff = 0.055) between samples (Figure 3C).

Phylum‐level gene abundance analysis identified Pseudomonadota, Bacteroidota, and Chloroflexota as the dominant phyla in Jinzihai Wetland (Figure 3E). The distribution of key microbial phyla exhibited habitat specificity: Acidobacteriota gene abundance was significantly higher in PB than SM (*p* < 0.05); Actinomycetota gene abundance was higher in SM, while Desulfobacterota, Planctomycetota, and Verrucomicrobiota gene abundances were higher in LS. LEfSe analysis further delineated habitat‐specific taxa (Figures A4, A5, A6, A7), revealing considerable phylogenetic divergence across habitats. Within the Archaea domain, Bathyarchaeia and *Methanothrix* were enriched in PB, whereas Aenigmatarchaeia and *Methanoregula* were characteristic of LS. Among Bacteria, Bacteroidia and Anaerolineales were enriched in SM; Acidobacteriota and Sphingomonadales were predominant in PB; and Desulfobacterota, Verrucomicrobiota, and Chromatiales were enriched in LS. For Eukaryota, Poaceae and Cyperaceae were associated with SM; Basidiomycota and *Dicrocoelium* were indicative of PB; biota such as Podocopida and *Ephemera* were enriched in LS. Notably, within the Viruses, only LS exhibited significant enrichment of specific taxa, such as Caudoviricetes, *Chloriridovirus*, and *Phaeovirus*.

Co‐occurrence network analysis identified carbon fractions, nitrogen fractions, TP, SWC, and pH as the environmental factors exhibiting the highest connectivity to species nodes (Figure 4A). Specifically, Acidobacteriota gene abundance correlated negatively with pH but positively with carbon fractions. Chloroflexota gene abundance correlated negatively with pH. Pseudomonadota gene abundance frequently correlated positively with nitrogen fractions and Fe content. Furthermore, key drivers of community structure varied among habitats (Figure 4B): In SM, community structure correlated significantly with pH (*r* = 0.58, *p* = 0.018). In LS, community structure correlated significantly with Ca (*r* = 0.20, *p* = 0.042). In PB, community structure correlated significantly with SWC (*r* = 0.40, *p* = 0.016), TC (*r* = 0.37, *p* = 0.029), and TOC (*r* = 0.35, *p* = 0.014).

### 3.3 Distribution Patterns of Functional Genes in Jinzihai Wetland

**FIGURE A1:**
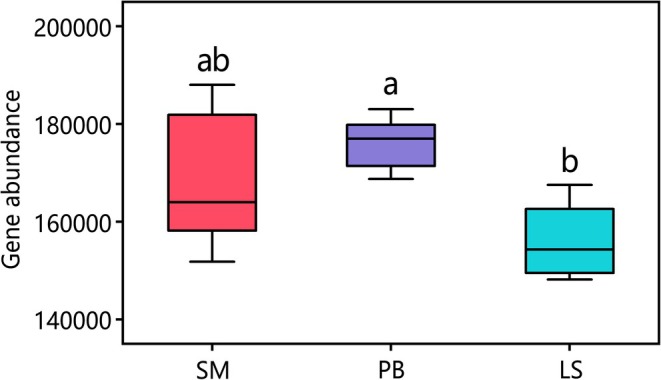
KEGG‐annotated metabolic pathway gene abundance across habitats. Central line and whiskers in each box represent the median and the min‐max values, boxes indicate interquartile range (25th–75th percentile). Different letters denote significant inter‐group differences (determined by two‐sided Wilcoxon test, FDR‐adjusted *p* < 0.05). LS, Lake sediments; PB, Peat bogs; SM, Sandy meadows.

PCoA of gene abundance revealed high compositional similarity among PB samples, while SM samples exhibited substantial variation (Figure 5A). Within LS, deep‐water samples (LS1‐3, > 5 m depth) showed homogeneous gene profiles, sharply contrasting with the shallow‐water sample (LS4, ~2 m depth), which displayed an intermediary gene profile between the deep‐water LS and the PB samples. COG annotation indicated enrichment in categories (Figure 5B): J (translation, ribosomal structure and biogenesis), K (transcription), L (replication/recombination and repair), M (cell wall/membrane/envelope biogenesis), and T (signal transduction mechanisms), alongside ubiquitous enrichment of metabolism‐associated genes. KEGG pathway analysis demonstrated significant enrichment of genes involved in metabolic pathways, biosynthesis of secondary metabolites, and ABC transporters (Figure 5C). Notably, metabolic pathway genes were significantly more abundant in PB than LS (*p* < 0.05), while SM displayed considerable heterogeneity (Figure A1).

CAZyme annotation identified glycoside hydrolases (GHs) as the dominant class, exhibiting significantly higher gene abundance in SM than LS (*p* < 0.05), with predominant families GH13, GH177, GH74, and GH23 (Figure 5D,E). Gene abundance of carbohydrate esterases (CEs) was enriched in SM and PB relative to LS (*p* < 0.05), with ce1 as the core family. Polysaccharide lyases (PLs) displayed significantly greater gene abundance in PB than LS (*p* < 0.05), primarily comprising PL22, PL9, PL12, and PL1. Carbon cycling analysis demonstrated distinct functional genetic composition among the three habitats (Figure 6A–D). SM was significantly enriched in genes related to photosynthetic carbon fixation, including those involved in photosystem I (*p* < 0.05), photosystem II (*p* < 0.01), cytochrome b6f complex (*p* < 0.01), and photosynthetic electron transport (*p* < 0.001). This habitat also showed significantly higher abundance of genes participating in disaccharide and glycolipid biosynthesis (*p* < 0.05), oxidation of formaldehyde (*p* < 0.01), and degradation of carbohydrates such as pectin and glycolipid (*p* < 0.05). Conversely, SM was notably depleted in genes associated with rTCA cycle (*p* < 0.01), CO_2_‐methane interconversion (*p* < 0.05), central methanogenic pathway (*p* < 0.001), and anaerobic oxidation of methane (AOM, *p* < 0.05). PB was significantly enriched in genes involved in the aceticlastic methanogenesis pathway (*p* < 0.01) and in the degradation of various carbohydrates, including disaccharides, cellulose, chitin, pectin, and peptidoglycan (all *p* < 0.05). In contrast, it was markedly depleted in key photosynthesis‐related genes, particularly those related to photosystem II (*p* < 0.01) and cytochrome b6f complex (*p* < 0.05). LS was significantly enriched in genes for CO_2_‐methane interconversion (*p* < 0.05), anaerobic oxidation of methane (*p* < 0.001), and central methanogenic pathway (*p* < 0.001). Conversely, it demonstrated a marked depletion of genes for photosynthetic electron transport (*p* < 0.001) and aceticlastic methanogenesis (*p* < 0.05), alongside a reduced abundance of pathways for carbohydrate synthesis and degradation.

Nitrogen cycling genes exhibited distinct enrichment patterns among the habitats (Figure 7A). SM was significantly enriched in the nitrite‐oxidizing gene *nxrA* (*p* < 0.001), the assimilatory nitrate reductase genes *nirA* (*p* < 0.05) and *narB* (*p* < 0.05), the dissimilatory nitrate reduction gene *nirD* (*p* < 0.05), the *ureA* gene (*p* < 0.05, encoding the urease alpha subunit), and the *gdh* gene (encoding glutamate dehydrogenase, GDH). PB showed significantly lower abundance of *napA* (*p* < 0.01) and *narG* (*p* < 0.01), but a higher abundance of the nitrification genes *hao* (*p* < 0.001) and *nxrA* (*p* < 0.01). LS exhibited enrichment in the dissimilatory nitrate reductase gene *napA* (*p* < 0.001), the denitrification genes *norB/C*, and the organic nitrogen mineralization gene *glsA*. Conversely, *nxrA* (*p* < 0.001) and *hao* (*p* < 0.05) were significantly less abundant in LS. Sulfur cycling genes also exhibited distinct enrichment patterns among the habitats (Figure 7B). SM showed enrichment of the assimilatory sulfate reduction genes *cysH/I/J*, as well as organic sulfur transformation genes *metB*, *comA/B*, *dddP*, and *dmdB/C/D*. LS exhibited enrichment of dissimilatory sulfate reduction (DSR) genes *aprA/B* and *dsrA/B*, tetrathionate reductase genes *ttrA/B*, and flavocytochrome *c* sulfide dehydrogenase gene *fccB*. PB displayed no significant enrichment of sulfur cycling genes relative to other habitats.

## 4 Discussion

### 4.1 Habitat Diversity Sustains Biodiversity in Wetland Ecosystems

Among the 49,574 species annotated in Jinzihai Wetland, 35% (17,129 species) were not shared across all habitats, indicating that habitat‐specialist species constitute a significant component of the wetland assemblage (Figure 3B). Specifically, SM exhibited the highest number of unique species (5919), reflecting its distinct saline‐alkali stressed community and pivotal role as a wetland‐desert ecotone sustaining regional biodiversity. Furthermore, terrestrial habitats (SM/PB) shared substantially more co‐occurring species (39,250) than SM‐LS or PB‐LS pairs, thus highlighting a fundamental aquatic‐terrestrial divide in community composition. β‐Diversity decomposition confirmed that community dissimilarity was driven primarily by replacement rather than richness difference (Figure 3C) (Legendre 2014). This replacement was clearly reflected in the differential enrichment of multiple taxonomic groups across habitats (Figures A4, A5, A6, A7), illustrating a process of bidirectional selection between organisms and environment. Consistent with this, α‐diversity patterns showed that all habitats supported high species richness, with no significant differences among them (Figure 3D). Collectively, these results demonstrate that habitat heterogeneity along environmental gradients drives community differentiation primarily through replacement. Therefore, preserving intra‐wetland habitat diversity is critical for safeguarding overall biodiversity by providing multidimensional niches for species with divergent ecological requirements.

### 4.2 Habitat Diversity Maintains Functional Diversity in Wetland Ecosystems

**FIGURE 8:**
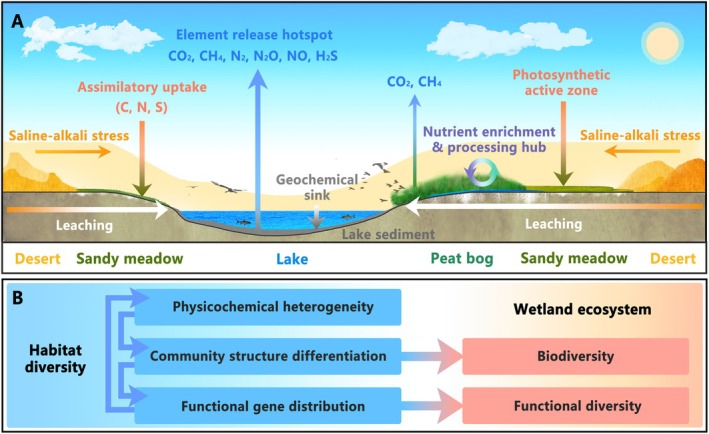
Mechanisms of ecosystem functioning maintenance in Jinzihai Wetland mediated by habitat diversity. (A) Functional specialization and synergy among habitats. (B) Habitat diversity framework and pathways for maintaining wetland ecosystem functioning.

Significant physicochemical gradients across Jinzihai Wetland habitats (Figure 2A–D) drive community differentiation mediated predominantly by species replacement (Figure 3C), thereby structuring divergent functional gene assemblages (Figure 5A). This hierarchical heterogeneity across elemental, community, and gene scales collectively constitutes the habitat diversity framework characteristic of plateau arid‐region wetlands (Figure 8B). Ultimately, this framework sculpts dominant functional differentiation among habitats while sustaining the diversity of ecosystem‐wide biogeochemical cycling processes (Figure 8A).

SM exhibits low SWC, low organic matter, and high salinity‐alkalinity stress (Figure 2A–D), with pH showing a significant correlation with the microbial community structure (Figure 4B). In this habitat, Actinomycetota genes are significantly enriched (Figure 3E), which cope with saline‐alkaline stress via intracellular compatible solute accumulation and ABC transporter reliance (Han et al. 2018). This finding corroborates KEGG‐annotated enrichment of ABC transporter pathway genes (Figure 5C), underscoring transmembrane transport as key for maintaining intracellular homeostasis. Carbon cycling features enhanced litter degradation, evidenced by enrichment of genes degrading disaccharides, cellulose, chitin, hemicellulose, pectin, and glycolipid (Figure 6D). This aligns with enrichment of GHs genes, particularly families GH13 (α‐amylase), GH177 (exo‐α‐sialidase), GH74 (xyloglucanase), and GH23 (glycoside hydrolase) (Figure 4E), pivotal for complex polysaccharide degradation (Nguyen et al. 2025). Consistent with reported GHs characteristics (Zhu et al. 2024), their abundance negatively correlates with TOC and TN but positively with pH, indicating adaptive resource acquisition strategies by microorganisms under nutrient stress involving specific enzymatic functions (Zhang et al. 2023). Furthermore, the low abundance of genes associated with the rTCA cycle, AOM, and central methanogenic pathways further supports the conclusion that SM is an aerobic environment, which intrinsically suppresses these characteristic anaerobic processes. Within this oxic setting, significant enrichment of photosynthetic electron transport and photosystems I/II genes suggests SM's low‐lying vegetation forms an effective light‐capturing interface (Figure 6A), designating it as a core area for photoautotrophic carbon fixation. This functional capacity is supported by enriched taxa (Figures A5, A6), including *Nodosilinea*, Cyperaceae, and Poaceae, which together form an effective light‐capturing interface. Notably, genes from *Nodosilinea* (a cyanobacterium known for oxygenic photosynthesis; Bozan et al. 2025) were markedly detected in SM, indicating its key role in supporting photosynthesis in Tibetan Plateau arid wetlands. Paradoxically, despite high photosynthetic carbon input, SM displays low TOC, highlighting poor organic carbon retention in its sandy soil, likely leaching organic matter to LS/PB habitats. Enrichment patterns of nitrogen cycling‐related genes also reveal SM's potential for enhanced biological assimilation (Figure 7A): the *nxrA* gene mediates nitrite oxidation to nitrate, subsequently assimilatorily reduced to ammonium via *narB* and *nirA*. The *gdh*‐encoded GDH then converts ammonium to glutamate for biosynthesis (Wang and Tan 2013). A similar assimilatory process characterizes sulfur cycling (Figure 7B): *cysH/I/J* genes, involved in the assimilatory sulfate reduction pathway (ASRP), drive sulfide production (Ploeg et al. 2001; Mansilla et al. 2000). Enriched *comA/B* genes convert sulfite to sulfopyruvate, yielding coenzyme M (Liu et al. 2012), while *metB* gene encodes cystathionine γ‐synthase to initiate methionine biosynthesis (Ferla and Patrick 2014). Notably, dimethylsulphoniopropionate (DMSP)‐related genes are also enriched, including *dddP* for cleavage to climate‐active dimethyl sulfide (DMS), and *dmdB/C/D* for the demethylation pathway (Curson et al. 2011). This enrichment may be linked to the stress conditions in SM, which may enhance DMSP synthesis (Haworth et al. 2017), highlighting a critical role for SM in sulfur cycling. Collectively, as a wetland‐desert ecotone, SM achieves high‐efficiency resource utilization through enhanced decomposition of complex organic matter, photoautotrophic carbon fixation, and nitrogen/sulfur assimilation, functioning as a pivotal hub driving assimilatory processes and functional responses to saline‐alkaline stress.

**FIGURE A2:**
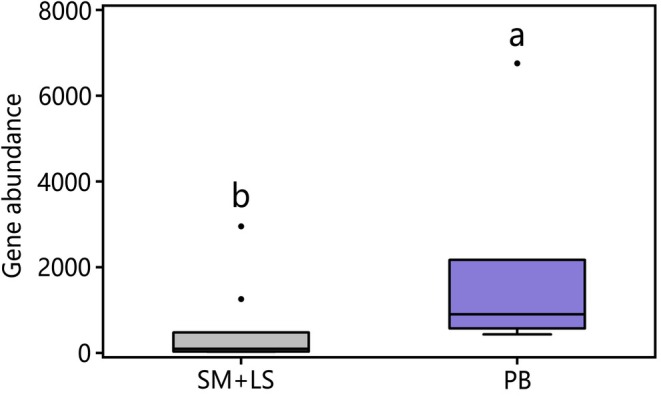
Gene abundance of archaeal *Methanosarcina* and *Methanothrix* across habitats. Central line and whiskers in each box represent the median and the min‐max values; boxes indicate interquartile range (25th–75th percentile). Different letters denote significant inter‐group differences (determined by Wilcoxon test, *p* < 0.05). PB, Peat bogs; SM + LS, Sandy meadows and lake sediments.

**FIGURE A3:**
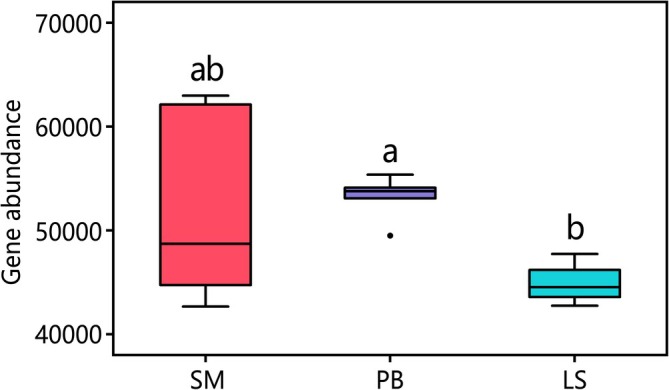
Total CAZyme gene abundance across habitats. Central line and whiskers in each box represent the median and the min‐max values; boxes indicate interquartile range (25th–75th percentile). Different letters denote significant inter‐group differences (determined by two‐sided Wilcoxon test, FDR‐adjusted *p* < 0.05). LS, Lake sediments; PB, Peat bogs; SM, Sandy meadows.

**FIGURE A4:**
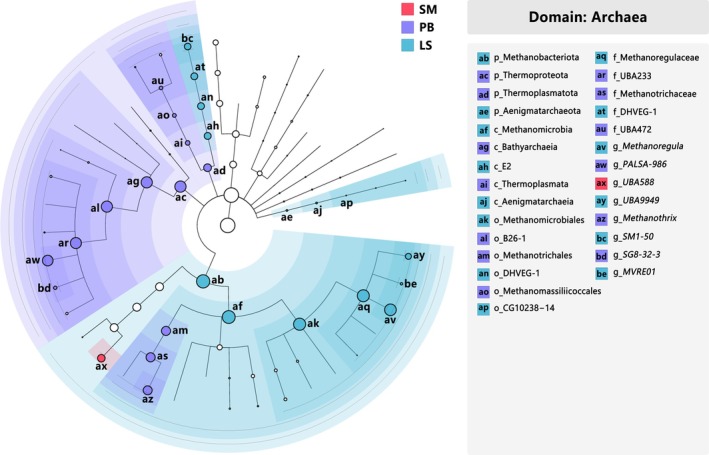
Key discriminatory taxa within the domain Archaea identified by linear discriminant analysis effect size (LEfSe) across multiple habitats. Taxonomic features were selected based on the following criteria: top 50 LDA scores (use_feature_num), top 100 abundance ranks (use_taxa_num), and statistical significance (*p* < 0.05). Node sizes represent gene abundance. Taxonomic ranks are indicated as: p‐phylum, c‐class, o‐order, f‐family, g‐genus.

**FIGURE A5:**
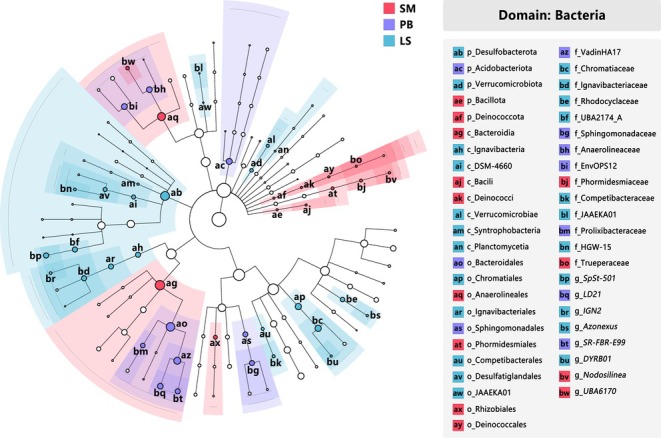
Key discriminatory taxa within the domain Bacteria identified by linear discriminant analysis effect size (LEfSe) across multiple habitats. Taxonomic features were selected based on the following criteria: Top 50 LDA scores (use_feature_num), top 150 abundance ranks (use_taxa_num), and statistical significance (*p* < 0.05). Node sizes represent gene abundance. Taxonomic ranks are indicated as: p‐phylum, c‐class, o‐order, f‐family, g‐genus.

**FIGURE A6:**
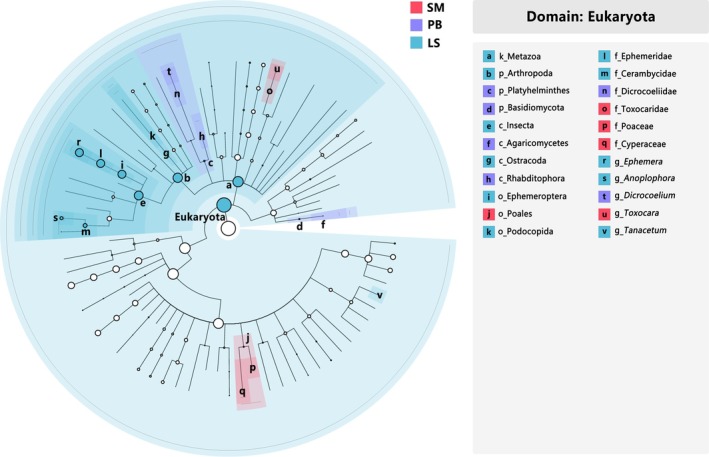
Key discriminatory taxa within the domain Eukaryota identified by linear discriminant analysis effect size (LEfSe) across multiple habitats. Taxonomic features were selected based on the following criteria: Top 100 LDA scores (use_feature_num), top 200 abundance ranks (use_taxa_num), and statistical significance (*p* < 0.05). Node sizes represent gene abundance. Taxonomic ranks are indicated as: k‐kingdom, p‐phylum, c‐class, o‐order, f‐family, g‐genus.

**FIGURE A7:**
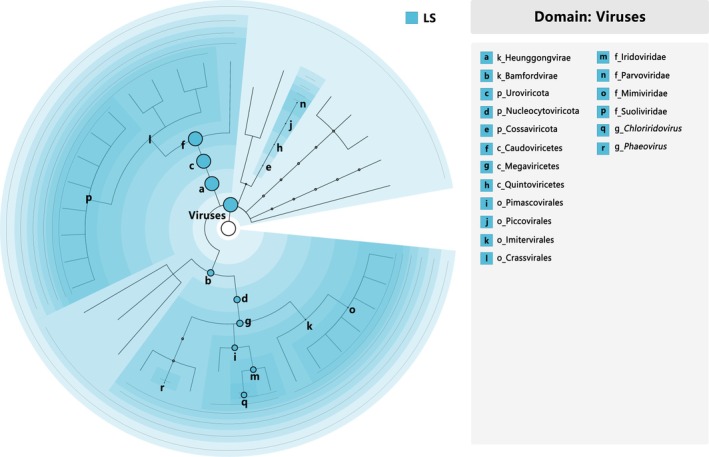
Key discriminatory taxa within the domain Viruses identified by linear discriminant analysis effect size (LEfSe) across multiple habitats. Taxonomic features were selected based on the following criteria: Top 100 LDA scores (use_feature_num), top 150 abundance ranks (use_taxa_num), and statistical significance (*p* < 0.05). Node sizes represent gene abundance. Taxonomic ranks are indicated as: k‐kingdom, p‐phylum, c‐class, o‐order, f‐family, g‐genus.

PB exhibits high SWC, organic matter content, and near‐neutral pH (Figure 2A–D). In this habitat, SWC serves a dual function: it correlates with organic matter, and both factors collectively associate with community structure (Figure 4B). Furthermore, SWC variation creates a mosaic of oxidative and reductive microzones that support diverse microbial taxa (Figures A4, A5, A6, A7). Specifically, the taxa enriched include aerobic Sphingomonadaceae, known for their role in hydrocarbon degradation (Kertesz et al. 2019); the anaerobic archaea Bathyarchaeia, involved in acetogenesis and lignin decomposition (Nesbø et al. 2024); and Acidobacteriota (Figure 3E), consistent with their adaptation to acidic conditions and complex organic matter degradation (Dedysh and Sinninghe‐Damste 2018), aligning with co‐occurrence network results (Figure 4A). The coexistence of these taxa corresponded with a significant enrichment of genes involved in carbohydrate degradation (Figure 6D), indicating an enhanced potential for organic matter utilization in PB. Consistently, regarding CAZymes, genes encoding PLs are enriched in PB (Figure 5D). Dominant families include PL22 (oligogalacturonate lyase), PL9 (pectate lyase), PL12 (heparan sulfate lyase), and PL1 (pectate lyase) (Figure 5E), suggesting a potential for enhanced degradation of plant/animal‐derived acidic polysaccharides. Additionally, as key mediators of acetoclastic methanogenesis, archaeal *Methanosarcina* and *Methanothrix* gene abundances are significantly higher in PB (*p* < 0.05; Figure A2) (Jetten et al. 1992). This is consistent with the observed increase in aceticlastic methanogenesis genes (Figure 5B), establishing PB as a methanogenic hotspot. Regarding energy metabolism, PB shows depletion of photosynthetic genes but significant enrichment of metabolic pathway genes (Figure 6A and Figure A3). These findings collectively support the view that PB sustains a chemotrophic system centered on organic acid metabolism, dependent on the degradation of diverse organic substrates. Nitrogen cycling in PB features distinct nitrification dynamics (Figure 7A): although *amo* gene absence, elevated *hao* and *nxrA* gene abundances suggest active nitrification, implying potential non‐classical AMO complexes or alternative hydroxylamine production pathways (Soler‐Jofra et al. 2021; Bayer et al. 2016). Concurrently, low abundances of nitrate‐reducing genes (*napA*, *narG*, and *nirA/B/D*) indicate reduced nitrate conversion, representing a microbial adaptive strategy to high‐NH_4_^+^‐N/low‐NO_3_^−^‐N conditions. No sulfur cycling genes are enriched in PB (Figure 7B), further supporting the notion that its redox status fluctuates between the oxidized SM and the reduced LS. In summary, as an environmentally dynamic habitat, PB mediates wetland nutrient enrichment and transformation through enhanced metabolism, polysaccharide degradation, aceticlastic methanogenesis, and nitrification.

LS is characterized by waterlogging‐induced hypoxia, exhibits enrichment of genes associated with anaerobic guilds (Figure 3E) and (Figures A4, A5, A6, A7), such as *Methanoregula*, Desulfobacterota, and Planctomycetota. *Methanoregula* is a typical H_2_/CO_2_‐utilizing methanogen (Zinder and Bräuer 2016) and exhibits co‐enrichment of its genes with central methanogenic pathway genes in LS, whereas aceticlastic pathway genes are significantly less abundant (Figure 6B), indicating that LS is a hotspot for H_2_/CO_2_‐dependent methanogenesis. Desulfobacterota mediate sulfur and carbon cycling, performing dissimilatory sulfate reduction (DSR) to convert sulfate to sulfide. This aligns with the enrichment of *aprA/B* and *dsrA/B* genes (Figure 7B), pointing to LS as a critical DSR habitat. Desulfobacterota also form syntrophic partnerships with anaerobic methanotrophic archaea (ANME) to mediate AOM (Deutzmann and Schink 2011), with corresponding AOM gene enrichment establishing LS as a key AOM site (Figure 6D). Consequently, the carbon cycle in LS is characterized by the dual role of methane (Figure 6B): mitigating methane release via AOM while concurrently generating methane. Planctomycetota, mediating nitrogen cycling through anaerobic ammonium oxidation (anammox) (Kündgen et al. 2025), show significant gene enrichment, indicating LS serves as a key habitat for anammox‐driven nitrogen removal. Furthermore, LS anoxia promotes nitrate reduction and denitrification, evidenced by enrichment of *napA*, *narG*, and *norB/C* genes (Figure 7A). Consequently, LS hosts coupled nitrogen removal processes: denitrification consumes nitrate to produce N_2_/N_2_O, while *glsA*‐mediated mineralized NH_4_^+^ is converted to N_2_ via anammox (Schwarz et al. 2022; Xu et al. 2021), establishing LS as a hotspot for nitrogen release. Moreover, LS functions as a regional sink that accumulates both genetic material and geochemical components. It serves as an enrichment site for genes of biota, such as *Ephemera* and Podocopida, while also accumulating genetic elements derived from viruses and certain terrestrial organisms (Figures A6, A7). Geochemically, LS acts as a repository for readily leachable elements (Fe, Mn; Figure 2D) and exhibits reduced carbohydrate metabolism, evidenced by significantly lower CAZyme gene abundance compared to terrestrial habitats PB (Figure A3). This suppressed carbon turnover aligns with the absence of organic matter degradation genes (Figure 6D). Additionally, upregulated *fccB* genes promote H_2_S oxidation to S^0^ (Figure 7B) (Yu et al. 2014), suggesting sulfur retention as a component of LS's geochemical sink function. In summary, as a low‐oxygen metabolic zone, LS regulates elemental release and serves as a geochemical sink in the wetland by enhancing key processes like AOM, H_2_/CO_2_‐dependent methanogenesis, anammox, and DSR while attenuating carbohydrate degradation and accumulating leached substances.

### 4.3 Habitat Diversity as a Mechanism for Sustaining Wetland Ecosystem Functioning

Based on the above analysis, we propose a mechanistic framework for the maintenance of habitat diversity in plateau arid‐region wetlands (Figure 8B): Marked physicochemical gradients drive community differentiation governed predominantly by replacement, thereby shaping the spatial distribution of functional genes. Biogeochemical processes mediated by these genes amplify habitat heterogeneity, establishing a self‐sustaining feedback loop. This habitat diversity underpins a distinct functional complementarity in microbially mediated biogeochemical cycling among the core habitats, forming a synergistic network supported by sustained microbial and functional gene diversity (Figure 8A). Specifically: SM functions as a saline‐alkali barrier and primary production zone, where enriched stress‐tolerant taxa buffer environmental stress, enhancing resistance while its sandy substrate facilitates dissolved nutrient export. PB serves as a hub for nutrient enrichment and transformation, where co‐accumulation of moisture and organic matter facilitates support for high‐intensity decomposition. LS acts as a terminal regulatory and burial zone, where anoxia drives key processes regulating carbon flux (via methanogenesis–AOM coupling), nitrogen removal (via anammox–denitrification synergism), and sulfur cycling (via DSR). In line with Biodiversity and Ecosystem Functioning (BEF) theory and the complexity‐stability hypothesis (Meng et al. 2021; Mougi 2020; Voris et al. 1980), we propose that the ecosystem multifunctionality emerging from this cross‐habitat functional complementarity constitutes a key mechanism underpinning wetland ecosystem stability. While the current DNA‐based metagenomic data are constrained to core habitats and reflect functional potential rather than in situ activity, our findings robustly demonstrate that habitat diversity sustains microbial functional gene diversity, thereby providing a mechanistic basis for ecosystem multifunctionality via its support of diverse biogeochemical processes. Future studies integrating Metatranscriptomics and finer‐scale spatial sampling will be essential to further validate these processes and more fully assess ecosystem stability in these arid wetlands.

## 5 Conclusions

Integrating metagenomic and geochemical analyses of the Jinzihai Wetland (Qaidam Basin, QTP), we demonstrate that habitat diversity sustains microbial functional gene diversity and underpins key biogeochemical processes in plateau arid‐region wetlands. Distinct physicochemical gradients across the SM, PB, and LS habitats drive microbial community differentiation primarily through species replacement, establishing a tightly coupled “environmental gradient—community assembly—functional divergence” framework. On the one hand, habitat diversity provides diverse ecological niches that support wetland biodiversity. On the other hand, habitats exhibit functional complementarity and synergy, collectively driving wetland biogeochemical cycling: SM habitats dominate assimilation and saline‐alkaline stress response; PB habitats regulate nutrient enrichment and transformation; LS habitats mediate element release and burial. Thus, habitat diversity sustains the functional diversity of biogeochemical cycling processes, thereby supporting the overall functioning of the ecosystem. Consequently, habitat diversity protection should be central to conservation and management of plateau arid‐region wetlands, securing the integrity of critical ecosystem functioning and ensuring long‐term ecosystem stability.

## Author Contributions

**Chenyu Wang:** conceptualization (equal), formal analysis (equal), investigation (equal), methodology (equal), visualization (equal), writing – original draft (lead). **Haicheng Wei:** conceptualization (lead), methodology (equal), writing – original draft (equal). **Ronglei Duan:** investigation (equal), methodology (equal). **Shi Jin:** formal analysis (equal), visualization (equal), writing – review and editing (equal). **Jinxin Wen:** investigation (equal), visualization (equal). **Hongyu Li:** investigation (equal), visualization (equal). **Aiying Cheng:** formal analysis (equal). **Chunliang Gao:** formal analysis (equal). **Hongpan Xue:** investigation (equal). **Yilin Hou:** visualization (equal).

## Funding

This study was supported by the National Natural Science Foundation of China, 42172019. Youth interdisciplinarity team of fundamental research, Qinghai Institute of Salt Lakes, Chinese Academy of Sciences, islJCTD‐2022‐1. Outstanding Youth Program of the Natural Science Foundation of Qinghai Province, 2023‐ZJ‐941J.

## Conflicts of Interest

The authors declare no conflicts of interest.

## Supporting information


**Data S1:** Database Annotation Results.xlsx.

**Data S2:** Data Analysis Results.xlsx.

## Data Availability

The metagenomic sequence data generated in this study are deposited in the NCBI Sequence Read Archive (SRA) database under the BioProject number PRJNA1297435. The original data are contained within the article and Supporting Information.

## Appendix A

**TABLE A1:** Coordinates and altitude of sampling sites.

Site	Longitude	Latitude	Altitude/m	Site	Longitude	Latitude	Altitude/m
SM1	97.8802 E	36.7176 N	2986	SM5	97.8795 E	36.7237 N	2988
SM2	97.8781 E	36.7194 N	2988	SM6	97.8817 E	36.7238 N	2988
SM3	97.8777 E	36.7213 N	2987	SM7	97.8874 E	36.7219 N	2991
SM4	97.8787 E	36.7224 N	2988				
PB1	97.8827 E	36.7120 N	2985	PB5	97.8869 E	36.7192 N	2988
PB2	97.8826 E	36.7166 N	2987	PB6	97.8848 E	36.7201 N	2987
PB3	97.8905 E	36.7190 N	2988	PB7	97.8845 E	36.7217 N	2988
PB4	97.8890 E	36.7204 N	2990				
LS1	97.8788 E	36.7205 N	2985	LS3	97.8810 E	36.7198 N	2985
LS2	97.8814 E	36.7220 N	2985	LS4	97.8818 E	36.7179 N	2985

**TABLE A2:** Physicochemical properties of analyzed samples.

SM habitat		SM1	SM2	SM3	SM4	SM5	SM6	SM7
pH	—	7.95	8.65	9.17	8.31	9.00	8.07	7.97
SWC	%	31.59	13.26	13.49	12.58	6.57	7.61	15.88
TC	wt%	1.91	0.92	1.18	0.85	1.02	0.91	0.98
TOC	wt%	0.45	0.05	0.09	0.05	0.08	0.04	0.18
LCP1	mg/L	54.92	18.20	27.54	29.50	17.70	18.64	36.23
LCP2	mg/L	30.31	19.18	26.74	22.91	19.46	15.87	30.22
RCP	wt%	0.08	0.03	0.03	0.04	0.05	0.03	0.06
TN	wt%	0.09	0.01	0.03	0.01	0.01	0.01	0.02
NH_4_^+^‐N	mg/kg	36.79	11.09	20.41	13.74	11.28	14.54	18.45
NO_3_^−^‐N	mg/kg	0.94	0.72	0.67	0.74	0.73	0.59	0.64
TDN	mg/kg	86.10	19.74	23.71	14.65	15.28	14.99	24.24
TS	wt%	0.15	0.27	0.13	0.21	0.24	0.21	0.07
SO_4_^2−^	mg/kg	287.51	194.43	187.38	40.86	204.14	91.53	258.42
TP	mg/kg	254.29	215.55	181.45	185.39	194.67	308.81	147.03
K	ppm	6652	7559	7002	7616	7513	7359	7420
Ca	ppm	25,902	13,393	22,673	14,475	18,435	15,678	17,229
Mn	ppm	172	190	242	127	204	290	141
Fe	ppm	8626	9541	9836	8048	8756	11,725	7498
Ba	ppm	297	300	335	324	386	292	337

PB habitat		PB1	PB2	PB3	PB4	PB5	PB6	PB7
pH	—	7.42	7.33	7.44	7.90	7.76	7.56	7.70
SWC	%	393.51	478.98	156.26	115.92	146.52	112.38	111.40
TC	wt%	12.77	16.68	5.42	4.35	7.37	3.08	3.30
TOC	wt%	12.35	16.08	3.84	2.53	5.90	2.01	2.79
LCP1	mg/L	326.41	888.95	144.60	101.80	156.80	96.72	100.50
LCP2	mg/L	125.70	353.62	98.02	52.59	120.90	53.33	47.96
RCP	wt%	2.12	11.72	2.88	1.23	3.80	0.72	2.03
TN	wt%	0.53	1.81	0.36	0.19	0.40	0.17	0.20
NH_4_^+^‐N	mg/kg	47.34	160.33	86.99	29.85	52.17	30.85	24.00
NO_3_^−^‐N	mg/kg	3.91	1.36	1.40	0.81	1.37	1.00	0.75
TDN	mg/kg	517.70	1025.60	366.10	73.00	239.30	104.40	63.85
TS	wt%	0.36	0.86	0.37	0.17	0.58	0.21	0.42
SO_4_^2−^	mg/kg	478.13	834.72	436.31	587.68	563.52	376.93	552.20
TP	mg/kg	418.96	924.89	598.32	423.03	432.26	335.01	351.75
K	ppm	1870	4632	6343	5253	4125	5662	6176
Ca	ppm	126,717	8836	34,519	49,301	56,628	30,606	21,107
Mn	ppm	704	130	379	447	498	606	167
Fe	ppm	6324	11,585	12,906	13,302	15,357	10,402	10,350
Ba	ppm	212	122	235	286	214	273	266

LS habitat SM habitat		LS1	LS2	LS3	LS4
pH	—	8.03	8.09	7.91	8.02
SWC	%	352.65	413.64	318.23	339.62
TC	wt%	2.60	1.72	3.65	3.11
TOC	wt%	1.18	0.43	1.38	0.31
LCP1	mg/L	73.91	33.81	70.36	41.47
LCP2	mg/L	67.35	31.96	56.73	32.42
RCP	wt%	0.70	0.22	0.61	0.07
TN	wt%	0.21	0.09	0.23	0.07
NH_4_^+^‐N	mg/kg	62.62	42.29	47.99	44.61
NO_3_^−^‐N	mg/kg	1.02	0.82	0.82	0.69
TDN	mg/kg	192.05	126.00	254.35	68.20
TS	wt%	0.31	0.13	0.32	0.11
SO_4_^2−^	mg/kg	699.37	477.08	237.61	165.51
TP	mg/kg	423.38	487.52	448.59	223.65
K	ppm	5489	7037	4585	3839
Ca	ppm	25,976	22,393	45,293	48,331
Mn	ppm	579	331	527	286
Fe	ppm	13,909	10,981	11,414	5938
Ba	ppm	239	295	251	200
